# Assessment of COVID-19 Morbidity and Mortality Among Patients With Autoimmune Diseases at King Abdulaziz University Hospital

**DOI:** 10.7759/cureus.52492

**Published:** 2024-01-18

**Authors:** Abdullah Altuwairqi, Abdulah H Ali, Abdulaziz A Alariefy, Sami Bahlas, Samer K AlZahrani, Essam W Zarei, Adnan E Alshaikh, Ahmed H Khan, Abdullah A Attar

**Affiliations:** 1 Department of Orthopaedics, King Abdulaziz University Hospital, Jeddah, SAU; 2 Department of Medicine, King Abdulaziz University Faculty of Medicine, Jeddah, SAU; 3 Department of Medicine/Rheumatology, King Abdulaziz University Faculty of Medicine, Jeddah, SAU

**Keywords:** systemic lupus erythematosus, rheumatoid arthritis, immunosuppressants, covid-19, autoimmune diseases

## Abstract

Background

The coronavirus disease 2019 (COVID-19) pandemic has raised significant concerns about the effects of the virus on patients with autoimmune diseases. Therefore, understanding the COVID-19 outcomes in this population is crucial for effective prevention and management.

Objective

This study aimed to investigate the association between autoimmune diseases and the severity of COVID-19 in terms of mortality and morbidity. Despite substantial advancements in pandemic-related research concerning COVID-19 and autoimmune diseases, there remain noteworthy gaps in our comprehension of this association, particularly due to limited investigations conducted in Saudi Arabia.

Methods

This was a retrospective record review of a tertiary center from January 2020 to January 2022. We included 120 patients, among whom 40 were diagnosed with autoimmune diseases, and 80 were age- and sex-matched controls. Afterward, we assessed their demographics, year of admission, intensive care unit (ICU) admission, health status, length of hospitalization, comorbidities, diagnosis of autoimmune diseases, and type of immunosuppressant therapy.

Results

Most of the included patients (mean age: 45.4 years) were females (65.8%). The ratio of non-autoimmune diseases to autoimmune diseases was 2:1, the mean length of hospitalization was 8.83 ± 8.16 days, and the median was seven days (interquartile range (IQR) = 3 to 11 days). Among them, 17.5% were admitted to the ICU and 10% died. The prevalence of autoimmune diseases was higher in women than in men (77.5%). The most common diseases were systemic lupus erythematosus (40%), rheumatoid arthritis (20%), and ankylosing spondylitis (10%). Regarding COVID-19 outcomes, ICU admissions were higher among patients with autoimmune diseases than those with non-autoimmune diseases (35% vs. 8.8%) (p<0.05). This trend was also observed in mortality, with a higher percentage of deaths among patients with autoimmune diseases (27.5% vs. 1.7%) (p<0.05). In addition, there were no significant differences between genders in terms of ICU admission, health status outcomes, or length of hospitalization among patients with autoimmune diseases (p>0.05). Notably, 25 patients were administered immunosuppressants. Of these, 18 (72%) used steroids only, while seven (28%) used both biological and steroid therapy. However, no significant associations were observed between the type of treatment used and outcomes such as ICU admission, health status at discharge, and length of hospitalization (p>0.05).

Conclusion

This study suggests that individuals with autoimmune diseases have more severe COVID-19 outcomes, as shown by ICU admission and mortality rates, than patients with non-autoimmune diseases. Furthermore, we observed that the use of immunosuppressant medications among patients with autoimmune diseases showed no noticeable effect on these outcomes.

## Introduction

Coronavirus disease (COVID-19) has rapidly spread globally since its outbreak in China in December 2019. On March 11, 2020, the World Health Organization declared COVID-19 a pandemic [1]. The infectious agent responsible for this disease is a newly identified strain of coronavirus associated with severe acute respiratory syndrome (SARS), known as severe acute respiratory syndrome coronavirus 2 (SARS-CoV-2) [2]. While affecting the respiratory system predominantly, it can also impact other systems, including the hematological, renal, cardiovascular, gastrointestinal, neurological, and immunological systems [3]. The COVID-19 symptom and prognosis spectrum includes mild symptoms with benign course that manifest as flu-like symptoms such as fever, fatigue, and cough, as well as severe symptoms such as multi-organ failure and acute respiratory distress syndrome (ARDS) [4,5]. Multiple factors have been linked to poor outcomes in COVID-19, including advanced age, smoking, and preexisting comorbidities such as diabetes mellitus (DM), hypertension (HTN), chronic pulmonary disease, and immunosuppression [6].

Autoimmune connective tissue diseases are several diseases that result from tissue damage or malfunction caused by the immune system's response to self-antigens. It could have an impact on numerous systems or a single organ. The etiology of autoimmune diseases is unclear; however, it is assumed that complex hereditary and environmental factors affect their development [7]. These diseases are a diverse collection of approximately 80 inflammatory disorders with a female predominance [8]. The most prevalent connective tissue diseases include rheumatoid arthritis (RA), systemic lupus erythematosus (SLE), Sjögren syndrome, scleroderma, and myositis [9,10]. The pathophysiology underlying these diseases is quite complicated, for this reason, the COVID-19 pandemic has raised significant concerns that individuals with autoimmune diseases are at higher risk of infection and complications, which are aggravated by the nature of their disease and/or the use of immunosuppressive medications [10,11].

Although research on the association between COVID-19 and autoimmune diseases is ongoing, multiple studies have provided valuable insights into the significant association between the two. Earlier research found that COVID-19 patients with autoimmune diseases have a higher risk of severe illness and worse outcomes than the general population [2-14]. Conversely, an American study revealed that COVID-19 patients with autoimmune diseases did not have a worse prognosis than matched controls [15]. Additionally, a Chinese study reported that the length of hospitalization and mortality rates were similar between autoimmune and non-autoimmune patients with COVID-19 [16].

Regardless of these findings, the field of COVID-19 associated with autoimmune diseases has made tremendous progress since the onset of the pandemic. However, it is essential to acknowledge that there are still significant gaps in understanding this relationship, particularly due to the limited research in Saudi Arabia. Another notable gap is the discrepancy in the results of several studies regarding the outcomes of COVID-19 in patients with autoimmune diseases. Therefore, we conducted this study to assess COVID-19 mortality and morbidity among patients diagnosed with autoimmune disease in Saudi Arabia.

## Materials and methods

Study design, setting and population

This retrospective study was conducted at King Abdul-Aziz University Hospital (KAUH) in Jeddah, Saudi Arabia, from May to August 2023. We included COVID-19 patients older than 18 years admitted to KAUH from January 2020 to January 2022. Among them, patients admitted to the intensive care unit (ICU) for reasons other than COVID-19 were excluded.

Data collection method

A pre-designed checklist was created to collect data from the KAUH database for all patient-related demographics (age, sex, nationality, and year of admission), outcomes (ICU admission, health status, and length of hospitalization (LHS)), comorbidities (diabetes mellitus, hypertension, asthma, ischemic heart disease, hypothyroidism, obesity, chronic kidney disease, heart failure, valvular heart diseases, dyslipidemia, obstructive sleep apnea, cancer, cerebrovascular accident, pulmonary hypertension, Cushing syndrome), diagnosis of any autoimmune disease and its type, and the usage of immunosuppressive treatment.

Statistical analysis 

Microsoft Excel 2016 was utilized for data entry, and statistical analysis was performed using SPSS Statistics version 21 (IBM Corp., Armonk, NY). Each patient with autoimmune diseases was binned to non-autoimmune diseases age- and sex-matched controls. A random number generator was used to select two controls for each patient with autoimmune diseases (matched 1:2 (case: control)). Descriptive statistics of the sociodemographic and clinical characteristic variables, including sex, age, nationality, date of admission, diagnosis of autoimmune disease, type of autoimmune disease, comorbidities, ICU admission, health status, and use of immunosuppressive drugs, were calculated to determine the frequency and percentages. Quantitative data were presented as mean and standard deviation (Mean ± SD). Mann-Whitney test was used to compare patients with autoimmune and non-autoimmune diseases according to demographics, ICU admission, health outcomes, admission year, and comorbidities. Moreover, bivariate analysis was done using the Chi-squared (χ2) tests to determine COVID-19 outcomes (ICU admission and health status) and sex differences between autoimmune disease patients and the matched controlled. Additionally, it was used to determine whether the usage of immunosuppressive treatment would affect COVID-19 outcomes among autoimmune disease patients. All p-values of <0.05 were defined as statistically significant.

Ethical approval

The Research Ethics Committee at KAUH, Jeddah, Saudi Arabia, granted ethical permission for the study (Ref: 272-23).

## Results

Patients' characteristics

The mean age of the included patients (n = 120) was 45.42 ± 15.63 years, the mean length of hospitalization was 8.83 ± 8.16 days, and the median was seven days (interquartile range (IQR) = 3 to 11 days). Most participants were female (65.8%) and of non-Saudi nationality (62.5%). Almost 17% (17.5%) were admitted to the ICU, out of whom 10% died. Most of the patients (69.2%) were admitted in 2020. The most common comorbidities were diabetes mellitus (DM) (32.5%), hypertension (HTN) (31.7%) and bronchial asthma (9.2%). Table 1 shows the patients' sociodemographic and clinical characteristics.

**Table 1:** Distribution of patients based on their demographics, ICU admission, health outcomes, admission year, and comorbidities Number of patients = 120; Age and length of hospitalization values are expressed as mean ± standard deviation, and others as numbers (n) and percentages (%); Any patient with a BMI of 30 or more is considered obese according to WHO classification. n: number, ICU: intensive care unit.

Variables	n (120)	%
Sex		
Male	41	34.2
Female	79	65.8
Age	45.42 ± 15.63	
Nationality		
Saudi	45	37.5
Non-Saudi	75	62.5
Date of admission (year)		
2020	83	69.2
2021	31	25.8
2022	6	5
Length of hospitalization (days)	8.83 ± 8.16	
ICU admission		
No	99	82.5
Yes	21	17.5
Health status at discharge		
Deceased	12	10
Hospitalized and alive	108	90
Comorbidities		
Diabetes mellitus	39	32.5
Hypertension	38	31.7
Bronchial asthma	11	9.2
Ischemic heart disease	9	7.5
Hypothyroidism	6	5
Obesity	6	5
Chronic kidney disease	5	4.2
Heart failure	4	3.3
Valvular heart diseases	2	1.7
Dyslipidemia	2	1.7
Obstructive sleep apnea	1	0.8
Colon cancer	1	0.8
Stroke	1	0.8
Secondary pulmonary hypertension	1	0.8
Cushing syndrome	1	0.8

Autoimmune disease patients 

**Figure 1:**
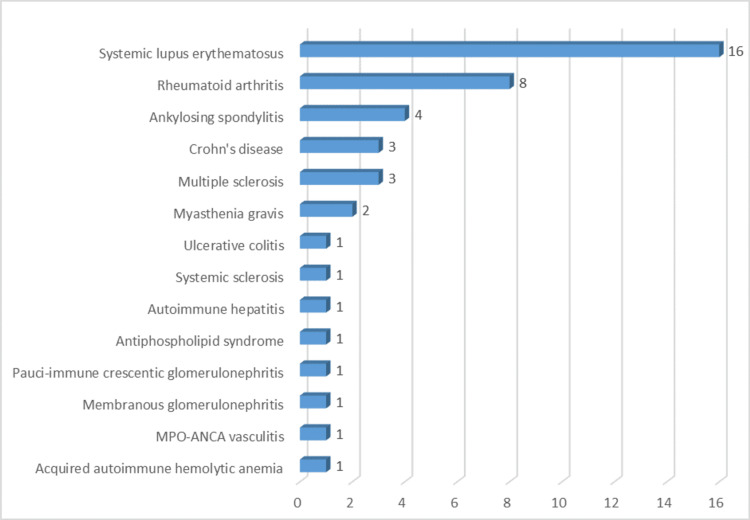
Frequency of types of autoimmune diseases among the diagnosed patients (Number = 40) MPO-ANCA vasculitis: Myeloperoxidase anti-neutrophil cytoplasmic antibody-associated vasculitis.

Table 2 illustrates a comparison between patients with and without autoimmune diseases according to demographics, ICU admission, outcome, year of admission, and comorbidities. Autoimmune disease prevalence was significantly higher among patients with comorbidities such as hypertension, hypothyroidism, and obesity (p<0.05). Among the patients included in this study, 40 were diagnosed with autoimmune diseases-the most common types were SLE (16 patients, 40%), RA (eight patients, 20%), ankylosing spondylitis (4 patients, 10%), and Crohn's disease (three patients, 7.5%), as shown in Figure 1.

**Table 2:** Comparison between patients with autoimmune and non-autoimmune diseases according to demographics, admission year, and comorbidities (Number of patients = 120) N.B.: * = Mann-Whitney test; n: number Notes: Age values are expressed as mean ± standard deviation, and others as numbers (n) and percentages (%).

Variables	Diagnosed with autoimmune disease		P-value
	No (n = 80)	Yes (n = 40)	
	n (%)	n (%)	
Age (years)	45.27 ± 15.8	45.7 ± 15.49	0.701
Sex			
Female	48 (60)	31 (77.5)	0.057
Male	32 (40)	9 (22.5)	
Nationality			
Non-Saudi	53 (66.3)	22 (55)	0.23
Saudi	27 (33.8)	18 (45)	
Date of admission (year)			
2020	57 (71.3)	26 (65)	0.028
2021	22 (27.5)	9 (22.5)	
2022	1 (1.3)	5 (12.5)	
Comorbidities			
Diabetes mellitus	22 (27.5)	17 (42.5)	0.098
Hypertension	20 (25)	18 (45)	0.026
Ischemic heart disease	7 (8.8)	2 (5)	0.462
Chronic kidney disease	3 (3.8)	2 (5)	0.747
Heart failure	3 (3.8)	1 (2.5)	0.719
Bronchial asthma	5 (6.3)	6 (15)	0.117
Hypothyroidism	1 (1.3)	5 (12.5)	0.008
Obesity	0 (0.0)	6 (15)	<0.001
Colon cancer	1 (1.3)	0 (0.0)	0.478
Valvular heart disease	1 (1.3)	1 (2.5)	0.614
Obstructive sleep apnea	0 (0.0)	1 (2.5)	0.156
Dyslipidemia	2 (2.5)	0 (0.0)	0.303
Stroke	1 (1.3)	0 (0.0)	0.478
Secondary pulmonary hypertension	0 (0.0)	1 (2.5)	0.156
Cushing syndrome	0 (0.0)	1 (2.5)	0.156

Of the examined patients with autoimmune conditions (n = 40), 25 (62.5%) were documented to use immunosuppressants. Among them, 18 (72%) were on steroids only, and seven (28%) were on a combination of biological treatment and steroid therapy. However, it should be noted that information about immunosuppressant therapy was not available for seven patients.

Relationship between autoimmune patients and COVID-19 outcomes

**Figure 2:**
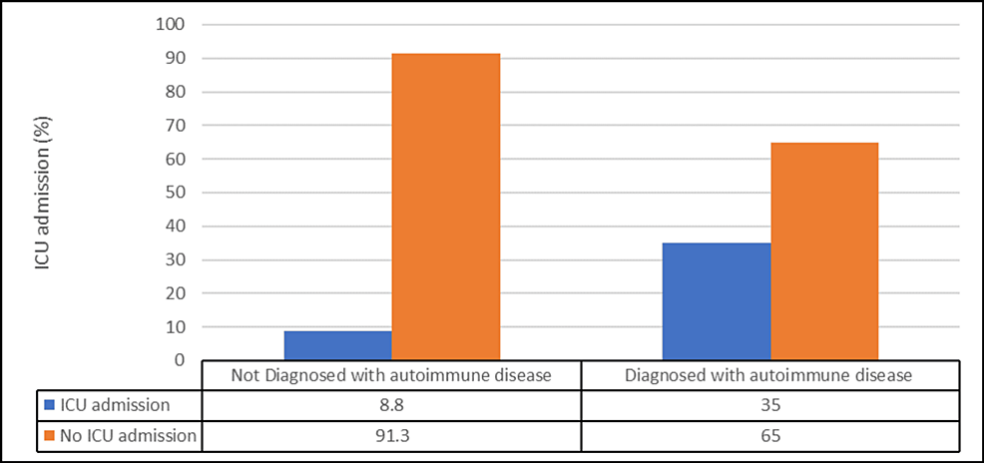
Comparison between patients with autoimmune and non-autoimmune diseases according to ICU admission (Number of patients = 120) (χ2 = 12.72, p-value = <0.001)

**Figure 3:**
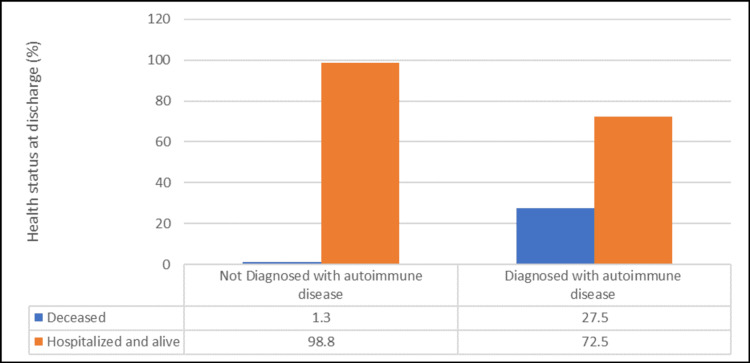
Comparison between patients with autoimmune and non-autoimmune diseases according to health status at discharge (Number of patients = 120)

Regarding COVID-19 outcomes, patients diagnosed with autoimmune diseases had a significantly higher prevalence of ICU admission (35% vs. 8.8%) (odd ratio (OR) 5.61, 95% CI 2.04-15.44, p<0.05), and their mortality rate was significantly higher (27.5% vs. 1.3%) (OR 29.96, 95% CI 3.7-242.48, p<0.05) (Table 3, Figures 2, 3). However, there was no noticeable gender difference in ICU admission, health status outcomes, or length of hospitalization (p>0.05) (Table 4).

**Table 3:** Comparison between patients with autoimmune and non-autoimmune diseases according to COVID-19 outcomes (Number = 120) Length of hospitalization values are expressed as mean ± standard deviation, and others as numbers (n) and percentages (%). n: number, ICU: intensive care unit

Variables	Diagnosed with autoimmune disease		χ^2^	p-value
	No	Yes		
	n (%)	n (%)		
Length of hospitalization (days)	7.75 ± 7.21	11 ± 9.52	1.67	0.094
ICU admission				
No	73 (91.3)	26 (65)	12.72	<0.001
Yes	7 (8.7)	14 (35)		
Health status at discharge				
Deceased	1 (1.3)	11 (27.5)	20.41	<0.001
Hospitalized and alive	79 (98.7)	29 (72.5)		

**Table 4:** Gender difference in COVID-19 outcome among patients diagnosed with autoimmune diseases (Number of patients = 40) Length of hospitalization values are expressed as mean ± standard deviation, and others as numbers (n) and percentages (%). n: number, ICU: intensive care unit

Variables	Gender		χ^2^	p-value
	Female	Male		
	n (%)	n (%)		
Length of hospitalization (days)	11.1 ± 9.98	10.67 ± 8.27	0.97	0.975
ICU admission				
No	22 (71)	4 (44.4)	2.15	0.142
Yes	9 (29)	5 (55.6)		
Health status at discharge				
Deceased	8 (25.8)	3 (33.3)	0.19	0.656
Hospitalized and alive	23 (74.2)	6 (66.7)		

Association between the usage of immunosuppressant therapy and COVID-19 outcomes

Finally, the mean length of hospitalization among patients with autoimmunity treated with immunosuppressants was slightly higher than that among those who were not; however, this relationship was not statistically significant, as shown in Table 5. Additionally, in further exploratory analyses, Table 6 shows that among patients with autoimmunity, there was no significant relationship between the type of treatment used and the length of hospitalization (days), ICU admission, and health status outcome (p>0.05).

**Table 5:** Relationship between the documented use of immunosuppressants among patients with autoimmune diseases and COVID-19 outcomes (Number of patients = 33) Length of hospitalization values are expressed as mean ± standard deviation, and others as numbers (n) and percentages (%). n: number, ICU: intensive care unit, COVID-19: coronavirus disease 2019.

Variables	Treated with immunosuppressant		p-value
	Yes	No	
	n (%)	n (%)	
	25	8	
Length of hospitalization (days)	11.56 ± 8.60	5.50 ± 4.53	0.068
ICU admission			
No	15 (60)	8 (100)	0.071
Yes	10 (40)	0 (0)	
Health status at discharge			
Deceased	8 (32)	1 (12.5)	0.394
Hospitalized and alive	17 (68)	7 (87.5)	

**Table 6:** Relationship between the type of treatment used among patients with autoimmune diseases and length of hospitalization (days), ICU admission, and health outcomes (Number of patients = 25) Length of hospitalization values are expressed as mean ± standard deviation, and others as numbers (n) and percentages (%). n: number, ICU: intensive care unit

Variables	Treated with immunosuppressant		p-value
	Steroids	Steroids and Biological Treatment	
	n (%)	n (%)	
Length of hospitalization (days)	12.50 ± 9.72	9.14 ± 4.33	0.246
ICU admission			
No	10 (55.6)	5 (71.4)	0.659
Yes	8 (44.4)	2 (28.6)	
Health status at discharge			
Deceased	7 (38.9)	1 (14.3)	0.362
Hospitalized and alive	11 (61.1)	6 (85.7)	

## Discussion

Autoimmune connective tissue diseases are chronic conditions that primarily affect women. RA, SLE, Sjögren syndrome, scleroderma, and myositis are the most prevalent connective tissue diseases. However, the pathophysiologies of these diseases are complex [17]. This study aimed to illustrate the morbidity and mortality of COVID-19 among patients diagnosed with autoimmune diseases and the effects of immunosuppressive therapy in Jeddah, Saudi Arabia.

In this study, the mean age of our patients was 45.4 ± 15.6 years, with females being the predominant gender (65.8%). This finding is consistent with the gender distribution reported in two other Saudi studies [17,18], reflecting the general trend of inflammatory autoimmune diseases being more prevalent among females.

Compared with the study by Alhowish et al. in Saudi Arabia [17], our patients' mean age was nearly similar at 48.3 years but younger than a study conducted in the United States, where the average age was reported as 63 years [19]. This variation could be attributed to the generally younger population in Saudi Arabia [20].

In our study of 120 patients, a subset of 40 individuals were diagnosed with autoimmune diseases, and their distribution revealed essential insights. SLE was the most prevalent autoimmune condition, accounting for 40% of all cases. Following SLE, RA represented the second most common autoimmune disease, affecting 20% of patients. Ankylosing spondylitis and Crohn's disease were also observed, with prevalence rates of 10% and 7.5%, respectively. The distribution of autoimmune diseases in our study aligns with the patterns observed in research conducted by D'Silva et al. [19]. However, their study, which is similar to ours, identified RA and SLE as the most common autoimmune diseases.

Furthermore, a striking contrast was observed in the prevalence of ICU admissions between patients with autoimmune diseases and healthy controls. Notably, 35% of patients diagnosed with autoimmune diseases required intensive care, in stark contrast to the 8.8% of ICU admissions in the healthy control group. This marked disparity underscores the heightened vulnerability of individuals with autoimmune diseases to COVID-19 and raises critical concerns for healthcare providers and public health strategists. Our findings align with those conducted by D'Silva et al. [19] and Arleo et al. [21], which reported similar trends in the increased risk of ICU admissions for COVID-19 patients with autoimmune diseases. We considered that the observed increase in ICU admissions among COVID-19 patients with autoimmune diseases may be attributed to various factors. One contributing factor is the underlying immune dysregulation that characterizes autoimmune diseases. This dysregulation can lead to a heightened inflammatory response during a viral infection, potentially contributing to more severe disease progression and the need for intensive care. This consistency in the results emphasizes the robustness of the association and reaffirms the significance of our findings.

However, it is essential to acknowledge that our results do not exist in isolation and that there are studies with contrasting outcomes. Some studies found no significant differences in ICU admission rates between COVID-19 patients with autoimmune diseases and healthy controls [15]. These discrepancies underscore the complexity of this relationship, indicating that variations in study populations, data collection methods, and specific autoimmune conditions may contribute to the differing results.

Our study revealed an apparent discrepancy in mortality rates between patients diagnosed with autoimmune diseases and healthy controls. An alarming 27.5% of the individuals with autoimmune diseases died from the virus, in sharp contrast to the minimal mortality rate of 1.3 % observed in the healthy control group. These findings underscore the profound vulnerability of individuals with autoimmune diseases to COVID-19, raising critical concerns for both healthcare providers and public health strategists. Moreover, we believe that this study offers a unique perspective on the challenges faced by individuals with autoimmune diseases during the COVID-19 pandemic. The amplified mortality rates observed in this patient population align with the broader understanding of how autoimmune diseases can impede the body's ability to respond effectively to viral infections. Similarly, intrinsic immune system dysregulation characterizing autoimmune conditions may play a pivotal role in accentuating disease severity, thereby contributing to increased mortality [22].

Notably, it is important to acknowledge that our study did not reveal similar outcomes to those in the existing literature. The absence of directly analogous studies may be attributed to the nascent nature of the COVID-19 pandemic and the limited availability of comprehensive data on this specific subgroup of patients. Thus, our research fills an imperative knowledge gap by highlighting a pivotal aspect of the intricate interplay between COVID-19 and autoimmune diseases.

Data from our study, which focused on 40 patients with autoimmunity, revealed that a significant proportion (62.5%) were documented as immunosuppressants. These findings highlight the prevalent use of these medications in patients with autoimmunity. Interestingly, within the subgroup using immunosuppressants, the majority (72%) relied solely on steroids, whereas a smaller but notable proportion (28%) used both biological treatment and steroid therapy. These findings were consistent with those of a study conducted by Alhowaish et al., which demonstrated a high prevalence of steroid use in the management of autoimmune patients [17]. However, there was no documentation for the immunosuppressant therapy status of seven patients. This underscores the importance of thorough and accurate medical records, as understanding the treatment landscape is crucial for assessing the impact of immunosuppression on COVID-19 outcomes in patients with autoimmune diseases.

Our investigation yielded compelling results, as we found no statistically significant differences in crucial clinical parameters, including the length of hospitalization, ICU admission rates, and mortality rates, between patients with autoimmune diseases who were prescribed immunosuppressants and those who were not. These findings suggest that immunosuppressive medications did not significantly affect key healthcare outcomes in our study. Interestingly, our results contradict those of a study conducted by Arleo et al., in which chronic steroid use was associated with increased disease severity and worse outcomes [21]. The disparity in these findings underscores the complexity of immunosuppressive therapies, indicating that their effects may vary depending on the specific medications used, underlying conditions, or patient population.

There are some limitations to this study. First, our study was limited by the available variables in the medical records, which may not have accounted for all potential confounders or effect modifiers that could have influenced the observed associations. Second, the generalizability of our findings may be restricted, as they are contingent on the specific patient population and the timeframe of the data collection, potentially limiting their applicability to broader populations or different periods. Furthermore, the study was conducted in a single hospital, which could potentially affect its statistical power, making it more challenging to detect significant associations. These limitations emphasize the need for a cautious interpretation of our results and underscore the importance of further prospective studies to provide a more comprehensive understanding of the connection between autoimmune diseases and COVID-19.

## Conclusions

This study revealed that COVID-19 mortality and morbidity rates were significantly higher among patients with autoimmune diseases, characterized by increased rates of ICU admission and mortality compared to patients without autoimmune diseases. While this study provides valuable insights, It is important to acknowledge that further research is required to fully comprehend the connection between COVID-19 outcomes and autoimmune diseases. Additionally, this study highlights the need for ongoing monitoring and evaluation of COVID-19 outcomes among patients with autoimmune diseases to improve our understanding and inform better clinical management strategies.
